# Effects of runt-related transcription factor 2 (*RUNX2*) on the autophagy of rapamycin-treated osteoblasts

**DOI:** 10.1080/21655979.2022.2037881

**Published:** 2022-02-16

**Authors:** Cheng Ren, Yibo Xu, Hongliang Liu, Zhimeng Wang, Teng Ma, Zhong Li, Liang Sun, Qiang Huang, Kun Zhang, Chengcheng Zhang, Yu Cui, Qian Wang, Yao Lu

**Affiliations:** a Department of Orthopaedic Surgery, HongHui Hospital, Xi’an Jiaotong University, Xi’an, Shaan’xi Province, China; b Yan’ an University, Yan’ an, Shaanxi Province, China; c Bioinspired Engineering and Biomechanics Center (BEBC), School of Life Science and Technology, Xi’an Jiaotong University, Xi’an, Shaan’xi Province, China

**Keywords:** Autophagy, rapamycin, RUNX2, p38MAPK signaling, osteoblasts

## Abstract

Autophagy occurs throughout the development and maturation of bone tissues and various types of bone cells and plays a vital role in osteoporosis progression. This study aimed to explore the role of runt-related transcription factor 2 (*RUNX2*) in osteoblast autophagy and its related molecular mechanisms. MC3T3-E1 cells were treated with different concentrations of rapamycin, and their viability was determined using a cell counting Kit-8 (CCK-8). The cells were then transfected with si-RUNX2 and *RUNX2* overexpression plasmids, and the viability of these rapamycin-treated cells was measured using CCK-8, while the expression of autophagy-related genes/proteins and osteoblast differentiation-related genes was determined using Western blotting and RT-qPCR. Finally, Alizarin red staining was used to observe osteoblast mineralization, and transmission electron microscopy was employed to detect autophagosomes in cells administered different treatments. Rapamycin significantly inhibited cell viability and promoted cell autophagy compared with the control (*P* < 0.05). Cells with RUNX2 knockdown and overexpression were successfully established. Further, *RUNX2* overexpression was found to significantly enhance the viability and osteoblast mineralization of rapamycin-treated cells and suppress cell autophagy. *RUNX2* overexpression also increased p-p38MAPK/p38MAPK levels and *ALP, OCN*, and *OSX* expression, and markedly downregulated Beclin-1, LC3-II/LC3-I, p62, ATG1, p-Beclin-1, and ATG5 levels (*P* < 0.05). However, the trends after *RUNX2* knockdown opposed those observed after *RUNX2* overexpression. *RUNX2* may regulate osteoblast differentiation and autophagy by mediating autophagy-related and osteoblast differentiation-related genes/proteins, as well as the p38MAPK signaling pathway.

## Graphical abstract

**Figure:**
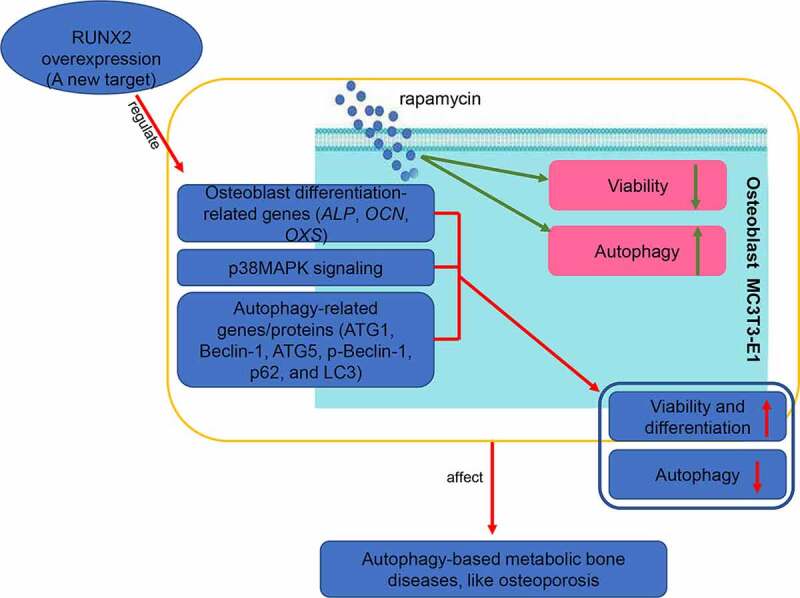

## Background

Bone remodeling is a tightly controlled mechanism that involves osteoclasts, osteoblasts, and osteocytes as primary factors [1]. Under normal circumstances, osteoblasts, osteoclasts, and osteocytes coordinate with each other to maintain homeostasis between bone resorption and bone formation, ultimately maintaining normal bone growth [2]. When bone metabolism is disordered, a series of metabolic bone diseases occur, including osteoporosis, rheumatic arthritis, osteolysis, and osteoarthritis [3]. Osteoporosis is the most common clinical condition and is mainly characterized by excessive bone resorption over bone formation, increased bone brittleness, destruction of the bone microstructure, low bone mass, easy fracture, and difficult healing [4]. According to statistics, 890,000 brittle fractures are caused by osteoporosis worldwide every year; in China, osteoporosis affects 40% of people older than 60 years, more than 50% of which are women [5]. Although age-related estrogen deficiency has long been considered a major cause of osteoporosis [6], its specific pathogenesis has not been fully elucidated.

Autophagy, a cellular survival mechanism under stress, can maintain cell homeostasis by wrapping misfolded proteins and aging organelles in autophagosomes for degradation [3]. Autophagy is reported to occur throughout the development and maturation of bone tissues and various types of bone cells, and has key effects on osteoporosis progression [7]. A study by Nollet et al. [8] revealed that the osteoblast mineralization ability was significantly reduced in cells with knockdown of autophagy-essential genes and osteoblast-specific autophagy-deficient mice. Their finding indicated that autophagy is closely associated with the mineralization process of osteoblasts and bone homeostasis. Another study revealed that oxidative stress caused by hydrogen peroxide (H_2_O_2_) induced autophagy and the apoptosis of osteoblast MC3T3-E1 cells in a time- and dose-dependent manner. According to the study, endoplasmic reticulum stress can relieve the oxidative damage of active oxygen in osteoblasts by inducing osteoblast autophagy [9]. These findings imply that the autophagy pathway plays a crucial role in the regulation of bone metabolic homeostasis, and abnormal autophagy may become a new pathogenesis of bone metabolic diseases.

Runt-related transcription factor 2 (*RUNX2*), a member of the RUNX family of transcription factors, is expressed in pluripotent mesenchymal cells, chondrocytes, and osteoblasts and regulates the proliferation, differentiation, and mineralization of chondrocytes and osteoblasts [10,11]. *RUNX2* is reported to enhance the proliferation of osteoblast progenitor cells by regulating fibroblast growth factor receptor 2 (*Fgfr2*) and fibroblast growth factor receptor 3 (*Fgfr3*), and stimulate the expression of bone matrix protein genes *in vitro*, such as collagen, type I, alpha 1 (*Col1a1*), secreted phosphoprotein 1 (*Spp1*), and fibronectin 1 (*Fn1*) [12]. Catheline et al. [13] reported that *RUNX2* overexpression could lead to more severe articular cartilage degeneration after traumatic joint injury, which might be due to the increased expression of matrix metallopeptidase 13 (*MMP13*) and the increased apoptosis of articular chondrocytes. Geng et al. [14] demonstrated that *RUNX2* is involved in osteoclast differentiation via the regulation of lysosome‑related genes that modulate receptor activator of nuclear factor-kappa B ligand (RANKL) expression. Additionally, fangchinoline is known to downregulate *RUNX2*, inhibit osteoblast apoptosis, and prevent bone loss by inducing autophagy in rats with osteoporosis [15]. Nonetheless, the effects of *RUNX2* on osteoblast autophagy and the related underlying mechanisms remain unclear.

Osteoblasts secrete a large amount of collagen, bone matrix, cytoactive factors, and enzymes to initiate the process of bone formation and control the generation, maturation, and activation of osteoclasts through a series of factors coupled with osteoclast [16]. Rapamycin is a special prophylactic agent of mammalian target of rapamycin (mTOR) and has been reported to participate in the autophagy pathway [17]. Therefore, in this study, we treated osteoblasts with different concentrations of rapamycin and investigated the role of rapamycin in osteoblast autophagy. Thereafter, we explored the role of *RUNX2* in osteoblast autophagy and its related molecular mechanisms. The results of this study will help us understand the roles of autophagy in osteoporosis and discover new potential targets for autophagy regulation.

## Materials and methods

### Cell culture

The mouse osteoblastic cell line, MC3T3-E1, was obtained from the Cell Bank of the Chinese Academy of Sciences (Shanghai, China). MC3T3-E1 cells were cultured in Dulbecco’s modified Eagle’s medium (DMEM, Thermo Fisher Scientific, Waltham, MA, USA) containing 10% fetal bovine serum (FBS, Thermo Fisher Scientific), 100 kU/L penicillin (Thermo Fisher Scientific), and 100 mg/L streptomycin (Thermo Fisher Scientific), and maintained in an incubator with 5% carbon dioxide at 37°C. The cells were passaged at 80%–90% confluence.

### Cell transfection

The si-RUNX2-(1/2/3)/si-negative control (si-NC) and RUNX2 overexpression plasmid (pcDNA3.1(+)-RUNX2)/empty vector (pcDNA3.1(+)) were designed and purchased from Yanzai Biotechnology (Shanghai) Co. Ltd (Shanghai, China). Cell transfection was performed as previously mentioned [18]. Briefly, MC3T3-E1 cells (1 × 10^4^ cells/well) were seeded in a 96-well plate and cultured overnight. The cells were then administered 10 nM rapamycin for 24 h, and the medium was replaced with serum-free medium. The cells were transfected with 4 μg pcDNA3.1(+)-RUNX2 /pcDNA3.1(+) and 4 μg si-RUNX2-(1/2/3)/si-NC using Lipofectamine 3000 (Thermo Fisher Scientific) according to the manufacturer’s protocol. After 6 h of transfection, the medium was replaced with complete medium. After culture for another 48 h, the total RNA from cells administered different treatments was isolated, and the expression of *RUNX2* was determined using reverse-transcription quantitative PCR (RT-qPCR) and Western blotting to assess cell transfection efficiency. Table 1 shows the RUNX2 sequences.

**Table 1.** The sequences of all primers

Primer	Sequence (5’-3’)
RUNX2-mF	ATGCTTCATTCGCCTCACAAA
RUNX2-mR	GCACTCACTGACTCGGTTGG
p38MAPK-mF	CTGACCGACGACCACGTTC
p38MAPK-mR	CTTCGTTCACAGCTAGGTTGC
LC3-II-mF	TTATAGAGCGATACAAGGGGGAG
LC3-II-mR	CGCCGTCTGATTATCTTGATGAG
Beclin-1-mF	ATGGAGGGGTCTAAGGCGTC
Beclin-1-mR	TCCTCTCCTGAGTTAGCCTCT
p62-mF	AGGATGGGGACTTGGTTGC
p62-mR	TCACAGATCACATTGGGGTGC
ALP-mF	CCAACTCTTTTGTGCCAGAGA
ALP-mR	GGCTACATTGGTGTTGAGCTTTT
OSX-mF	GGAAAGGAGGCACAAAGAAGC
OSX-mR	CCCCTTAGGCACTAGGAGC
OCN-mF	AGGAGGGCAATAAGGTAGT
OCN-mR	CATAGATGCGTTTGTAGGC
COL1A1-mF	GCTCCTCTTAGGGGCCACT
COL1A1-mR	CCACGTCTCACCATTGGGG
GAPDH-mF	GGTGAAGGTCGGTGTGAACG
GAPDH-mR	CTCGCTCCTGGAAGATGGTG

### Cell viability assay

MC3T3-E1 cells (1 × 10^4^ cells/well) were seeded in 96-well plates and cultured overnight. Thereafter, different concentrations of rapamycin (0, 10, 20, 50, and 100 nM) were administered to the cells. After 24 and 72 h of incubation, cell viability was measured to determine the optimal concentration of rapamycin and the optimal culture time.

MC3T3-E1 cells with different treatments were harvested and mixed with 10 μL of Cell Counting Kit-8 reagent (CCK-8, Beyotime Biotechnology). After 2.5 h of incubation, a microplate reader was used to measure the absorbance at 450 nm, and cell viability was calculated.

### RT-qPCR

Total RNA of cells administered different treatments was extracted using the RNAiso Plus assay kit (Trizol, Takara Biomedical Technology Co. Ltd., Beijing, China) following the manufacturer’s instructions. The quality and concentration of total RNA were assessed using a microplate reader (OD260/OD280) and 1% agarose gel electrophoresis. The isolated RNA was reverse transcribed into cDNA using the PrimeScriptTM II 1st Strand cDNA Synthesis kit (TaKaRa). The temperature protocol used for reverse transcription was 37°C for 60 min, and 85°C for 5 s. The sequences of all primers are displayed in Table 1, and qPCR was performed using SYBR Premix EX Taq (2x, Thermo Fisher Scientific, USA). The following cycling program was used for qPCR: 50°C for 3 min, 95°C for 3 min, followed by 40 cycles of 95°C for 10s and 60°C for 30s. Glyceraldehyde-3-phosphate dehydrogenase (*GAPDH*) served as the housekeeping gene, and the relative mRNA levels of *RUNX2*, p38 MAP kinase (*p38MAPK*), microtubule-associated protein 1 light chain 3 alpha (*LC3-II), COL1A1*, heat shock 90-like protein (*p62*), alkaline phosphatase (*ALP), Beclin-1*, Sp7 transcription factor (*OSX*), and bone gamma-carboxyglutamate protein (*OCN*) were calculated using the 2^−ΔΔCt^ method [19].

### Western blot

Total protein was extracted from cells using RIPA protein lysis buffer (Beyotime), and protein concentrations were determined using a BCA assay kit (Wuhan Boster Biological Technology, Ltd., Wuhan, China) following the manufacturer’s recommendations. Protein samples (20 μg) were separated by 10% sodium dodecyl sulfate polyacrylamide gel electrophoresis (SDS-PAGE), transferred to polyvinylidene fluoride (PVDF) membranes, and then blocked with 5% skimmed milk at 37°C. After 2 h of blockage, the membranes were incubated with anti-RUNX2 antibody (1:1000, ProteinTech Group, Inc.), anti-p38MAPK anti-body (1:1000, ProteinTech Group, Inc.), anti-phosphorylation (p)-p38MAPK antibody (1:1000, Cell Signaling Technology), anti-LC3 antibody (1:1000, Cell Signaling Technology), anti-Beclin-1 antibody (1:1000, Cell Signaling Technology), anti-p-Beclin-1 antibody (1:1000, Cell Signaling Technology), anti-p62 antibody (1:1000, ProteinTech Group, Inc.), anti-Autophagy-related protein 1 (ATG1) antibody (1:1000, Cell Signaling Technology), anti-Autophagy-related protein 5 (AGT5) antibody (1:1000, Cell Signaling Technology), and anti-GAPDH (1:5000, ProteinTech Group, Inc.) at 4 °C overnight. After three washes, the membranes were incubated with secondary antibodies (goat anti-mouse or goat anti-mouse IgG, 1:10,000; Jackson ImmunoResearch Laboratories, Inc.) at 37°C for 2 h. Following another round of washing, an ECL assay kit (Beyotime) was used to visualize the protein bands. Image-Pro Plus software (6.0, Media Cybernetics Imaging Technologies Inc., USA) was used to quantify the protein bands.

### Alizarin red staining

The osteogenic mineralization [20] of cells was determined using an Alizarin Red Staining Kit (Servicebio, Wuhan, China). First, osteogenic mineralization induction solution was prepared using β-glycerophosphate (10 mM), ascorbic acid (50 μg/mL), and dexamethasone (100 nM). Thereafter, MC3T3-E1 cells with different treatments were incubated in the osteogenic mineralization induction solution for 21 days. After removing the medium and three washes with phosphate buffer saline (PBS), the cells were fixed with 4% paraformaldehyde for 10 min and stained with Alizarin red S solution for 30 min. After removing the staining solution and washing with distilled water, the stained cells were observed and photographed under a microscope.

### Observation of autophagosomes by transmission electron microscopy (TEM)

Cells with different treatments were collected and washed twice with PBS. Thereafter, 2.5% glutaraldehyde was added to fix the cells, which were cultured overnight. After three washes with PBS, the cells were treated with 1% osmium tetroxide, fixed at 4°C for 30 min, washed with PBS, and dehydrated with different concentrations of acetone (in order of 50%, 70%, 90% and 100%) for approximately 10 min each. Following the removal of acetone, the sample was embedded in embedding medium and incubated at room temperature overnight. Thereafter, 1-μm sections were cut and stained with sodium acetate-lead citrate trihydrate. Finally, the autophagosomes were observed and analyzed using TEM (JEOL Ltd., Tokyo, Japan).

### Statistical analysis

Statistical analyses were performed using GraphPad Prism 5 software (GraphPad Software, San Diego, CA, USA). Data are expressed as mean ± standard deviation (SD). Prior to the statistical analysis, Shapiro-Wilk test was used to perform normal distribution. Thereafter, one-way analysis of variance (ANOVA) followed by Tukey’s method or Dunnett’s T3 method was carried out to compare more than two groups, while Student’s t-test was used for comparisons between two groups. Statistical significance was set at P < 0.05.

## Results

### Selection of the optimum concentration of rapamycin

**Figure 1.**
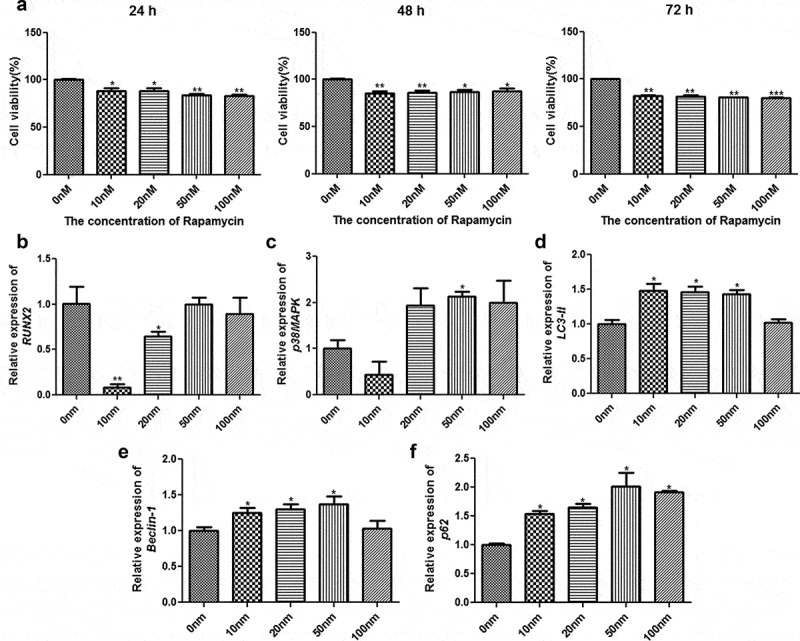
Selection of the optimum concentration of rapamycin. (a) Viability of MC3T3-E1 cells treated with different concentrations of rapamycin based on cell counting Kit-8. The mRNA expression levels of runt-related transcription factor 2 (*RUNX2*) (b), p38 map kinase (*p38 MAPK*) (c), microtubule-associated protein 1 light chain 3 alpha (*LC3-II*) (d), *Beclin-1* (e), and heat shock 90-like protein (*p62*) (f). *: *P* < 0.05, compared with 0 nm rapamycin. **: *P* < 0.01, compared with 0 nm rapamycin.

Different concentrations of rapamycin were used to treat MC3T3-E1 cells for different times. Cell viability was also measured using CCK-8 to select the optimum rapamycin concentration and culture time. After culture for 24 h, 48 h, and 72 h, the viability of cells treated with rapamycin was found to be significantly inhibited relative to those that were not subjected to rapamycin treatment (*P* < 0.05, Figure 1a). After 72 h of culture, a significant difference was found in the viability of MC3T3-E1 cells between cells treated with different concentrations of rapamycin (10 nm, 20 nm, 50 nm, and 100 nm) and those that were not subjected to rapamycin treatment (*P* < 0.01, Figure 1a). Therefore, MC3T3-E1 cells treated with rapamycin for 72 h were selected for RT-qPCR analysis.

The expression levels of *RUNX2, p38 MAPK, LC3-II, Beclin-1*, and *p62* were determined using RT-qPCR. Compared with cells without rapamycin treatment, those treated with 10 nm and 20 nm rapamycin had significant downregulation of *RUNX2* (*P* < 0.05); however, no significant difference in *RUNX2* expression was found among the cells treated with 0, 50, and 100 nm rapamycin (*P* > 0.05, Figure 1b). The expression of p38 MAPK was found to be significantly higher in the group of cells treated with 50 nm rapamycin than the other groups of cells (*P* < 0.05, Figure 1c). After treatment with 10, 20, and 50 nm rapamycin, the expression levels of *LC3-II* and *Beclin-1* were upregulated in MC3T3-E1 cells compared with those found in cells that were not subjected to rapamycin treatment (*P* < 0.05, Figure 1d,e). Additionally, *p62* expression was markedly upregulated in cells treated with rapamycin compared to MC3T3-E1 cells without rapamycin treatment (*P* < 0.05, figure 1f). Based on the cell viability and RT-qPCR results, MC3T3-E1 cells treated with 0, 10, and 50 nm rapamycin for 72 h were selected for Western blot analysis.

### Rapamycin induces osteoblasts MC3T3-E1 autophagy by regulating related genes

**Figure 2.**
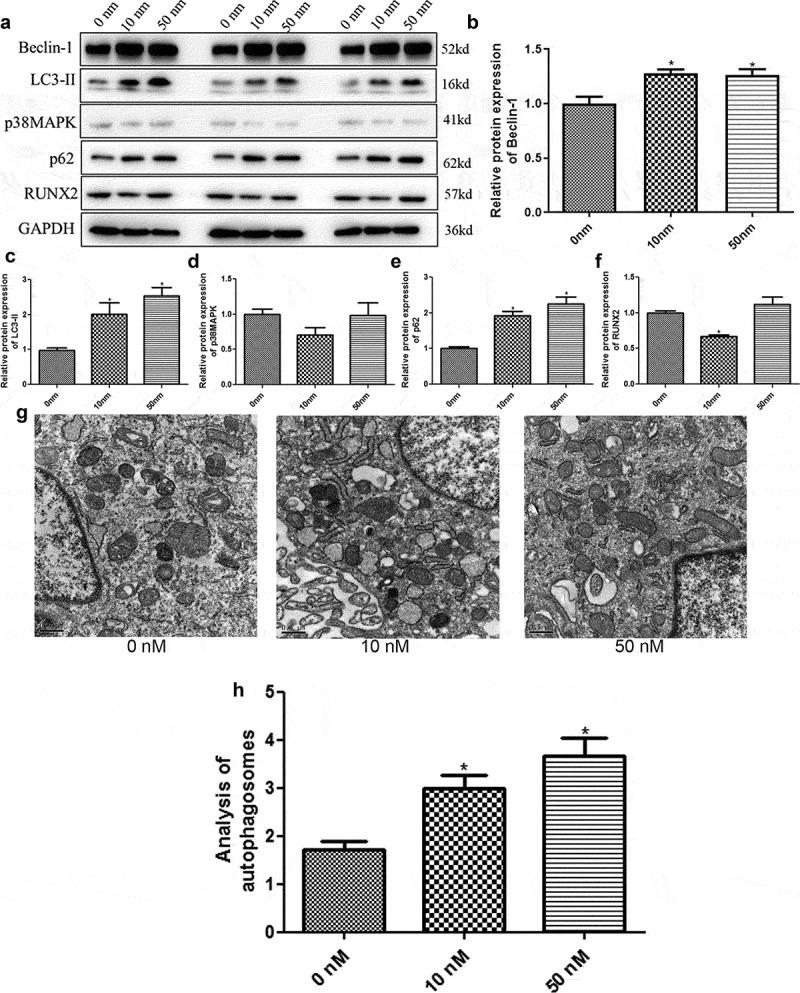
Rapamycin induced osteoblast MC3T3-E1 autophagy by regulating related genes. (a) The protein bands of related genes determined by Western blot. (b) Gray analysis of Beclin-1. (c) Gray analysis of microtubule-associated protein 1 light chain 3 alpha (LC3-II). (d) Gray analysis of p38 map kinase (p38 MAPK). (e) Gray analysis of heat shock 90-like protein (p62). (f) Gray analysis of runt-related transcription factor 2 (RUNX2). (g) Autophagosomes in cells treated with 0 nm, 10 nm, and 50 nm rapamycin treatment. (h) Quantitative analysis of autophagosomes. *: *P* < 0.05, compared with 0 nm rapamycin.

Western blotting was used to detect the protein expression of Beclin-1, p38MAPK, p62, LC3-II, and RUNX2 in different groups. Evidently, no significant difference was found in Beclin-1 protein expression between the cells treated with 10 nm and 50 nm rapamycin (*P* > 0.05), whereas Beclin-1 expression was significantly upregulated in rapamycin-treated cells compared to the cells without rapamycin treatment (*P* < 0.05, Figure 2a,b). The protein expression levels of -II and p62 in rapamycin-treated cells were higher than those in control cells (*P* < 0.05, Figure 2a,c,e). Further, p38 MAPK expression was not significantly different among the cells treated with 0, 10, and 50 nm rapamycin (*P* > 0.05, Figure 2a,d). The trend in RUNX2 protein expression in the different groups was found to be similar to that of Beclin-1 protein expression (Figure 2a,f).

The effects of rapamycin on autophagy in MC3T3-E1 cells were evaluated by detecting autophagosomes using TEM. Compared to the cells without rapamycin treatment, 10 nm and 50 nm rapamycin induced the production of autophagosomes by MC3T3-E1 cells (Figure 2g). Quantitative analysis revealed that the number of autophagosomes was significantly higher in MC3T3-E1 cells treated with 10 nm and 50 nm rapamycin than in control cells (*P* < 0.05, Figure 2h). These results indicate that rapamycin could induce autophagy in osteoblast MC3T3-E1 cells.

### Successful establishment of MC3T3-E1 cells with RUNX2 knockdown or overexpression

**Figure 3.**
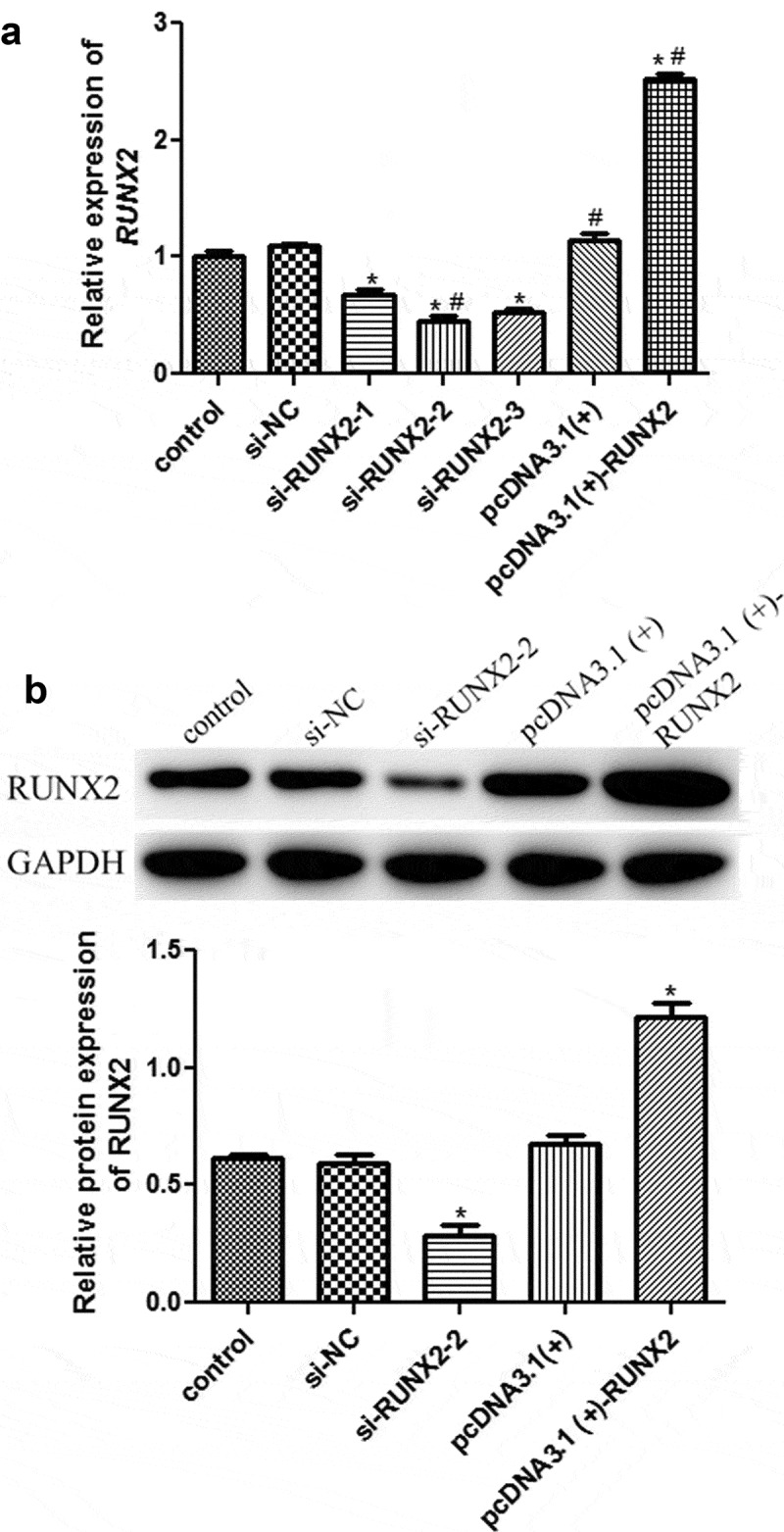
Cell transfection efficiency analysis based on runt-related transcription factor 2 (RUNX2) expression. (a) The mRNA expression of *RUNX2* after transfection using real-time quantification PCR (RT-qPCR). (b) The protein expression of RUNX2 based on Western blot. *: *P* < 0.05, compared with the control group. ^#^: *P* < 0.05, compared with si-RUNX2-1.

To explore the roles of *RUNX2* in cell autophagy, the *RUNX2* overexpression plasmid and si-RUNX2-(1/2/3) were transfected into rapamycin-treated MC3T3-E1 cells, and cell transfection efficiency was evaluated by RT-qPCR and Western blotting. No significant difference in RUNX2 expression was found among the control, si-NC, and pcDNA3.1 (+) groups (Figure 3). Compared with the control group, si-RUNX2-1/2/3 markedly downregulated the mRNA expression of *RUNX2* (*P* < 0.05), whereas the mRNA expression of *RUNX2* was significantly upregulated in the pcDNA3.1 (+)-RUNX2 group (*P* < 0.05, Figure 3a). Western blotting further verified the RT-qPCR results (Figure 3b). The RT-qPCR and Western blot results indicated that MC3T3-E1 cells with RUNX2 knockdown and overexpression were successfully established.

### RUNX2 overexpression enhances the viability of rapamycin-treated MC3T3-E1 cells by regulating autophagy-related genes/proteins and osteoblast differentiation-related genes

**Figure 4.**
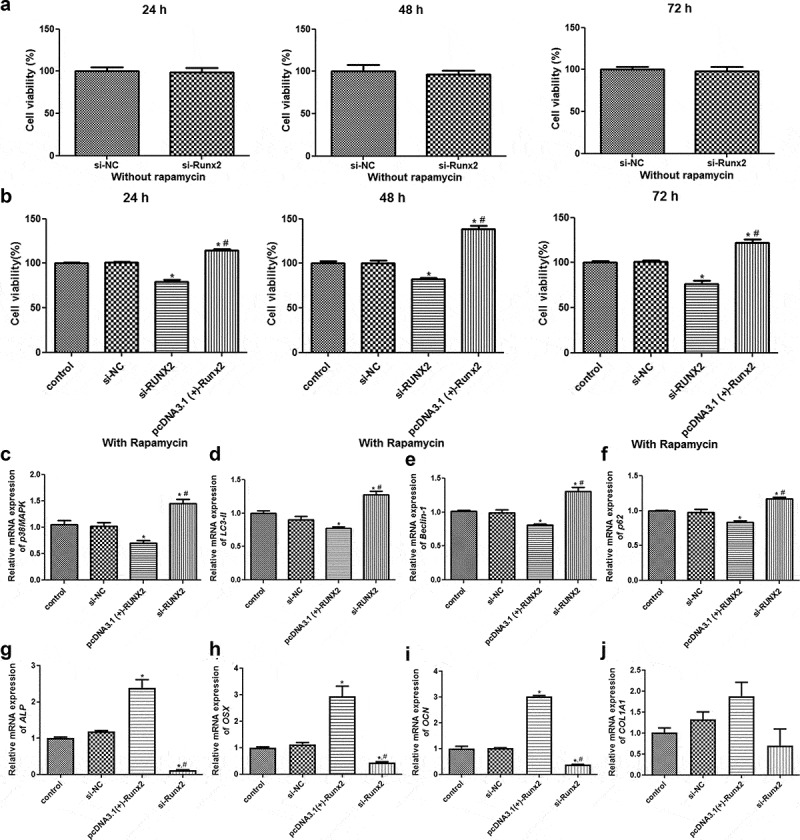
Runt-related transcription factor 2 (RUNX2) overexpression enhanced the viability of rapamycin-treated MC3T3-E1 cells by regulating autophagy-related genes and osteoblast differentiation-related genes. (a) Viability of MC3T3-E1 cells without rapamycin treatment after transfection with si-NC and si-RUNX2. (b) Viability of rapamycin-treated MC3T3-E1 cells with *RUNX2* overexpression or knockdown. *: *P* < 0.05, compared with the control group; ^#^: *P* < 0.05, compared with si-RUNX2 group. The mRNA expression levels of p38 map kinase (*p38 MAPK*) (c), microtubule-associated protein 1 light chain 3 alpha (*LC3-II*) (d), *Beclin-1* (e), heat shock 90-like protein (*p62*) (f), alkaline phosphatase (*ALP*) (g), Sp7 transcription factor (*OSX*) (h), bone gamma-carboxyglutamate protein (*OCN*) (i) and collagen, type I, alpha 1 (*COL1A1*) (j). *: *P* < 0.05, compared with the control group; ^#^: *P* < 0.05, compared with pcDNA3.1(+)-RUNX2 group.

The effects of *RUNX2* on the viability of rapamycin-treated MC3T3-E1 cells were determined using CCK-8. CCK-8 revealed that after different culture times, no significant difference in viability was found between the MC3T3-E1 cells transfected with si-NC and si-RUNX2, and between the cells treated with rapamycin and those with rapamycin treatment transfected with si-NC (*P* > 0.05, Figure 4a,b). In addition, compared to control cells, RUNX2 knockdown significantly inhibited the viability of rapamycin-treated MC3T3-E1 cells at 24, 48, or 72 h, whereas *RUNX2* overexpression markedly enhanced their viability (*P* < 0.05, Figure 4b).

To further investigate the molecular mechanisms by which RUNX2 affects autophagic cells, the mRNA expression levels of *p38MAPK, LC3-II, Beclin-1, p62*, and osteoblast differentiation-related genes (*ALP, COL1A1, OSX*, and *OCN*) were determined by RT-qPCR, and the protein expression levels of p38MAPK, p-p38MAPK, LC3-I, LC3-II, Beclin-1, p-Beclin-1, p62, AGT1, and AGT5 were determined by Western blotting. RT-qPCR revealed no obvious differences in the expression of related genes between the control and si-NC groups (*P* > 0.05, Figure 4c–J). Compared to the control group, the mRNA expression levels of p38 MAPK, LC3-II, Beclin-1, and p62 were significantly downregulated in the pcDNA3.1 (+)-RUNX2 group (*P* < 0.05), and significantly upregulated in the si-RUNX2 group (*P* < 0.05, Figure 4c–f). Further, the mRNA expression levels of ALP, OSX, and OCN were significantly increased after *RUNX2* overexpression compared to those in the control group (*P* < 0.05), and markedly reduced after *RUNX2* knockdown (*P* < 0.05, Figure 4g–i). However, *COL1A1* mRNA expression in the control, si-NC, pcDNA3.1(+)-Runx2, and si-Runx2 groups was not significantly different (*P* > 0.05, Figure 4j).

**Figure 5.**
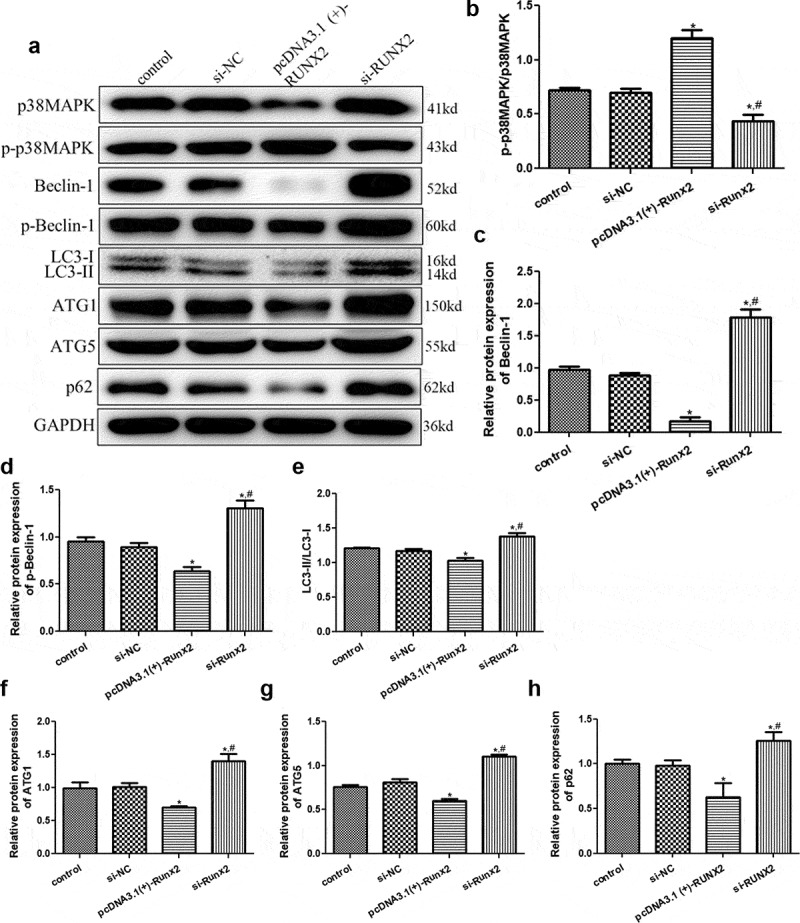
Effects of runt-related transcription factor 2 (*RUNX2*) on the protein expression levels of autophagy-related proteins and p38 MAPK signaling pathway. (a) The protein bands of related proteins based on Western blot. The protein levels of p-p38 map kinase/ p38 map kinase (p-p38 MAPK/p38 MAPK) (b), Beclin-1 (c), p-Beclin-1 (d), microtubule-associated protein 1 light chain 3 alpha (LC3-II/LC3-I) (e), Autophagy-related protein 1 (ATG1) (f), Autophagy-related protein 5 (ATG5) (g), and heat shock 90-like protein (p62) (h). *: *P* < 0.05, compared with the control group; ^#^: *P* < 0.05, compared with pcDNA3.1(+)-RUNX2 group.

The protein expression levels of p38MAPK, LC3-II, p-p38MAPK, Beclin-1, p-Beclin-1, LC3-I, p62, AGT1, and AGT5 were measured by Western blotting. The expression of related proteins was not significantly different between the control and si-NC groups (*P* > 0.05, Figure 5). Further, the level of p-p38 MAPK/p38 MAPK was significantly increased in cells after *RUNX2* overexpression (*P* < 0.05) and significantly decreased in the cells with RUNX2 knockdown compared to the control (*P* < 0.05, Figure 5a,b). The expression levels of Beclin-1 and p-Beclin-1 detected by Western blotting were consistent with those of Beclin-1 mRNA expression by RT-qPCR (Figure 5a,c,d). Additionally, the trend of LC3-II/LC3-I levels in the different groups opposed that of the p-p 38 MAPK/p38 MAPK levels (Figure 5a,e). For ATG1 and ATG5, their expressions were significantly lower in the cells with RUNX2 overexpression (*P* < 0.05) than control cells, and significantly higher in cells with RUNX2 knockdown than control cells and cells with RUNX2 overexpression (*P* < 0.05, Figure 5a,F,G). The trend of p62 protein expression in the different groups based on Western blotting was similar to that of p62 mRNA expression by RT-qPCR (Figure 5a,h).

### RUNX2 overexpression promotes osteoblast mineralization and inhibits autophagy in rapamycin-treated MC3T3-E1 cells

**Figure 6.**
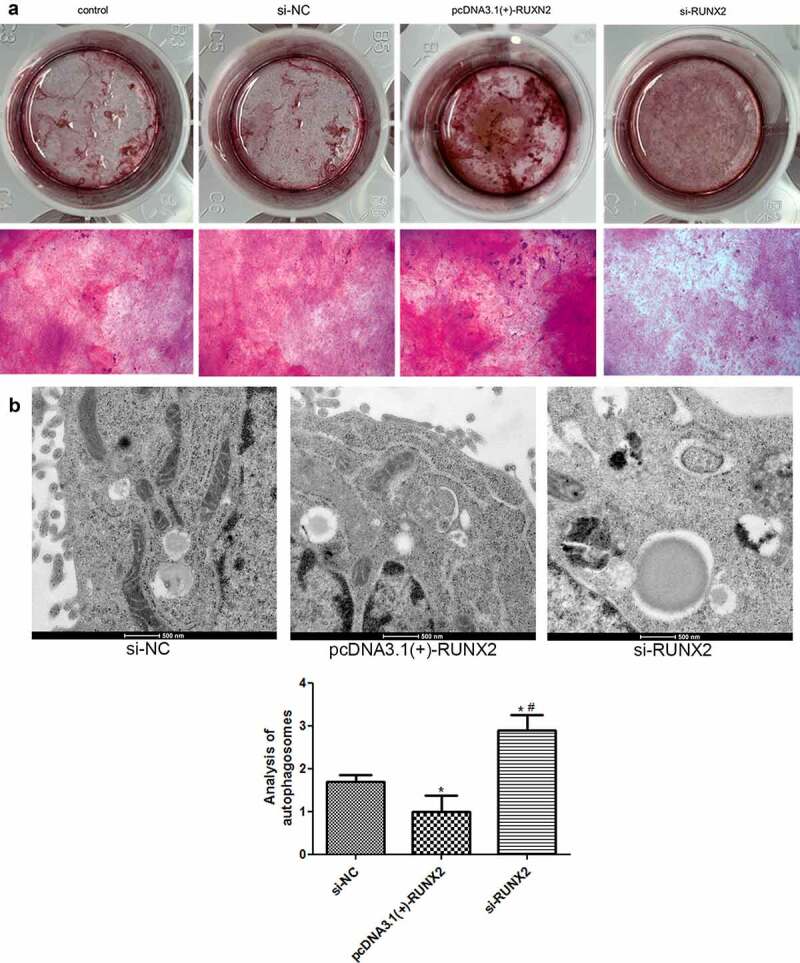
Effects of runt-related transcription factor 2 (*RUNX2*) on osteoblast mineralization and autophagy in rapamycin-treated MC3T3-E1 cells. (a) The osteoblast mineralization of MC3T3-E1 cells with different treatments determined by Alizarin red staining. (b) Autophagosomes in cells administered different treatments using transmission electron microscopy. *: *P* < 0.05, compared with the si-NC group; ^#^: *P* < 0.05, compared with the pcDNA3.1(+)-RUNX2 group.

To further explore the role of *RUXN2* in osteoblast mineralization and autophagy, alizarin red staining and TEM were performed. After *RUNX2* overexpression, the cells displayed deeper alizarin red staining; however, in the si-RUNX2 group, lighter Alizarin red staining was observed (Figure 6a). These findings indicate that *RUNX2* overexpression could promote osteoblast mineralization of MC3T3-E1 cells caused by rapamycin, and vice versa.

The number of autophagosomes was significantly reduced in the RUXN2 overexpression group (*P* < 0.05) compared to the si-NC group, and evidently increased in the si-RUNX2 group (*P* < 0.05, Figure 6b). These findings suggest that *RUNX2* overexpression could decrease the degree of autophagy caused by rapamycin in MC3T3-E1 cells, while *RUNX2* knockdown could enhance autophagy induced by rapamycin.

## Discussion

Autophagy mediates the onset and development of a variety of bone diseases, including osteoporosis [21]. *RUNX2* is a primary factor in osteoblast differentiation and has been reported to play an essential role in the development of osteoarthritis [22]. However, its role in the autophagy of osteoblasts remains unclear. In this study, MC3T3-E1 cells were treated with different concentrations of rapamycin. Rapamycin was found to inhibit cell viability and promote cell autophagy. In fact, 10 nm rapamycin significantly downregulated the expression of RUNX2 and upregulated p62, LC3-II, and Beclin-1. *RUNX2* overexpression and knockdown cells were successfully constructed and treated with rapamycin (10 nm). Compared to the control group, *RUNX2* overexpression significantly enhanced the viability of rapamycin-treated cells, promoted osteoblast mineralization of MC3T3-E1 cells, and suppressed cell autophagy. *RUNX2* overexpression also increased p-p38 MAPK/p38 MAPK levels, upregulated *ALP, OSX*, and *OCN* mRNA expression levels, and downregulated Beclin-1, p-Beclin-1, ATG1, ATG5, p62, and LC3-II/LC3-I. The trends after *RUNX2* knockdown opposed those observed after *RUNX2* overexpression. These results implied that 10 nm rapamycin could promote cell autophagy, and *RUNX2* may affect the viability, osteoblast mineralization, differentiation, and autophagy of rapamycin-treated cells by regulating the expression of autophagy-related genes and proteins (LC3-II, LC3-1, ATG1, p-Beclin-1, ATG5, p62, and Beclin-1), osteoblast differentiation-related genes (*ALP, OSX*, and *OCN*), and pathways (p38 MAPK).

The mTOR signal transduction pathway is the main regulator of cell growth and metabolism. Accordingly, dysregulation of the mTOR signaling pathway has been implicated in many human diseases, such as diabetes, cancer, obesity, and nervous system diseases [23,24]. Rapamycin is a specific inhibitor of the mTOR pathway and has been shown to be useful in the treatment of some disease [25]. A previous study indicated that microautophagy in mammals is a pathway for the delivery of cytosine to multivesicular bodies, and rapamycin is an activator of microautophagy in mammalians [26]. Our study revealed that the viability of MC3T3-E1 cells was inhibited after treatment with 10 nm rapamycin and the autophagy level was increased. Additionally, 10 nm rapamycin significantly downregulated Beclin-1 and RUNX2 and upregulated LC3-II and p62 levels. Sotthibundhu et al. [27] demonstrated that rapamycin induced autophagy and the adhesion of pluripotent stem cells, and Beclin-1 and LC3-II levels were increased with higher autophagy. Another study demonstrated that rapamycin could regulate the activation of macrophages by repressing the NLRP3 inflammasome-p38 MAPK-NFκB pathway in an autophagy and p62 manner [28]. Taken together, we speculate that 10 nm rapamycin may suppress the viability of osteoblasts and promote autophagy by regulating the expression of Beclin-1, p62, RUNX2, and LC3-II.

*RUNX2* has been reported to have vital effects on osteoblast differentiation and bone formation [29]. Tosa et al. [30] revealed that postpartum *RUNX2* loss results in low bone mass and accumulation of fat cells in the bone tissues of mice. We found that RUNX2 overexpression promoted osteoblast mineralization and the differentiation of MC3T3-E1 cells through alizarin red staining and upregulated the expression of *ALP, OSX*, and *OCN. ALP* and *OCN* are two key osteoblast-related marker genes. Among them, *ALP* plays a key role in early osteogenesis and hydrolyzes all types of phosphates to promote cell calcification and maturation; however, *OCN* accelerates the progression of later osteogenesis by binding with minerals [31]. Ma et al. [32] indicated that resveratrol alleviated the suppressive effect of lipopolysaccharides on osteoblast differentiation by upregulating ALP, OCN, OPN, and RUNX2. *OSX* plays a crucial role in the regulation of chondrocyte and osteoblast biology, and a previous study showed that MgCl_2_ could facilitate osteoblast differentiation by activating the p38 signaling pathway and upregulating OSX and RUNX2 [33]. Taken together, we speculate that *RUNX2* may promote osteoblast mineralization and the differentiation of MC3T3-E1 cells by upregulating osteogenic differentiation-related genes (*ALP, OSX*, and *OCN*).

Li et al. [34] revealed that vitamin K2 could stimulate MC3T3‑E1 osteoblast differentiation and mineralization through autophagy induction. Therefore, we further investigated the role of *RUNX2* in the autophagy of MC3T3‑E1 osteoblasts. In the current study, cells with *RUNX2* overexpression and knockdown were successfully established and treated with 10 nm rapamycin. *RUNX2* overexpression increased cell viability and inhibited autophagy. In addition, *RUNX2* overexpression increased p-p38MAPK/p38MAPK level, as well as downregulated Beclin-1, LC3-II/LC3-I, ATG1, ATG5, p-Beclin-1, and p62. Their tendency after transfection with si-RUNX2 opposed that after transfection with the *RUNX2* overexpression plasmid. p38MAPK, an important MAPK in stress signaling, can control key processes of cell homeostasis, including proliferation, self-renewal, death, and differentiation [35]. Elango et al. [36] showed that collagen peptide can upregulate the proliferation and differentiation of bone marrow mesenchymal stem cells and activate *RUNX2* to upregulate osteogenesis through the p38MAPK signaling pathway. LC3-II and LC3-I have been shown to be autophagosomal markers in mammals, and LC3-II/L3-I has been used to study autophagy in various diseases [37]. Beclin-1, a mammalian lineal homologous of yeast ATG6, is a core component of autophagy and plays a central role in autophagy regulation through the activation of VPS34 [38]. P-Beclin-1 has been reported to be a marker during the early stages of autophagy and has been used to evaluate cell autophagy [39]. ATG1 and ATG5 are closely associated with cell autophagy, and their downregulation results in lower autophagy level [40]. P62, another autophagy-related gene, has a crucial effect on autophagy and cell apoptosis, and is critical for cancer therapy [41]. Cao et al. [42] revealed that quercetin increased Beclin-1 and LC3-II/I levels and promoted autophagy of oxidized low-density lipoprotein-treated RAW264.7 macrophages. Another study reported that high levels of miR-101-3p suppressed autophagy in hepatocellular carcinoma cells by reducing LC3-II and Beclin-1 expression and increasing p62 expression [43]. Based on these findings and our results, *RUNX2* overexpression can be inferred to downregulate autophagy-related genes/proteins (LC3-II/LC3-I, p-Beclin-1, ATG1, ATG5, Beclin-1, and p62), and inhibit the autophagy of MC3T3-E1 cells via the p38MAPK signaling pathway, thereby playing a role in the occurrence and development of metabolic bone diseases.

## Conclusions

In conclusion, rapamycin (10 nm) successfully induced autophagy in osteoblasts and inhibited their viability. *RUNX2* overexpression may facilitate cell viability and osteoblast differentiation by regulating the expression of osteoblast differentiation-related genes (*ALP, OCN*, and *OXS*). Additionally, *RUNX2* overexpression may suppress autophagy in rapamycin-treated cells through the p38MAPK signaling pathway and by mediating autophagy-related genes and proteins (ATG1, Beclin-1, ATG5, p-Beclin-1, p62, and LC3-II/LC3-I). Our findings provide new insights into the treatment of autophagy-based metabolic bone diseases, with *RUNX2* as a potential target and RUNX2/p38MAPK as the underlying pathway.

## Data Availability

The dataset used and/or analyzed during the current study are available from the corresponding author on reasonable request.
